# Editorial: Phenomenological psychopathology: who, what and how? An analysis of key figures, advancements and challenges

**DOI:** 10.3389/fpsyg.2025.1644046

**Published:** 2025-08-14

**Authors:** Susi Ferrarello, Francesca Brencio, Valeria Bizzari, Magnus Englander

**Affiliations:** ^1^Department of Philosophy and Religious Studies, California State University, East Bay, Hayward, CA, United States; ^2^School of Psychology, University of Birmingham, Birmingham, United Kingdom; ^3^Husserl Archive, KU Leuven, Leuven, Belgium; ^4^Department of Social Work, Malmö University, Malmö, Sweden

**Keywords:** phenomenology, health, Husserl, Heidegger, psychiatry

The world of psychiatry is a world situated within a broader medical context. Hence its mainstream model of science is concerned with an investigation into mental states being analogous to a medical model and a clinical practice that begins with a diagnosis followed by treatment. The founders of phenomenological psychiatry, in the beginning of the twentieth century, tried to challenge the shortcomings of such a natural scientific approach. Already in 1913, Karl Jaspers emphasized the relationship between the individual and the community and how a science and practice of psychopathology could never abstract from the intersubjective context (Jaspers, 1997, p. 710). Among the pioneers were also Eugene Minkowski, Erwin Straus, Ludwig Binswanger, Medard Boss, and many others. Later in the twentieth century, a critique of modern psychiatry was raised in the work of, for example, Michel Foucault, R. D. Laing, Rollo May, and Franco Basaglia, among others. Lately, we have witnessed how research in phenomenological psychology has been able to contribute to a recovery-oriented psychiatry in the work of, for example, Larry Davidson. We have also seen a transcultural and social approach to phenomenological psychiatry in research conducted by, for example, Laurence Kirmayer. Contemporary phenomenological psychopathology and psychiatry is still flourishing as can be seen in efforts of, for example, Thomas Fuchs, Irwin Yalom, Josef Parnas, Giovanni Stanghellini, Matthew Broome, Mads Gram Henriksen, among many others. Despite all this work, psychopathology and the practice of psychiatry remains associated with the medical model, predominantly ruled by a natural scientific approach to human problems.

In modern psychology, the study of psychopathology seems to be on par with biomedical psychiatry and can be seen in the specialization in clinical and abnormal psychology. The dominant role of cognitive-behavioral therapy within mainstream psychology is grounded in a theoretical model of the psyche that seems like a mere epiphenomenon of the biomedical model.

Psychiatry appears to follow the principle of “descriptions, not interpretations,” which guided the Kraepelinian model. This approach tends to dismiss any alternative viewpoints as unscientific or lacking evidence. The field generally treats mental illnesses as concrete, physical things (a process called reification), rather than as complex psychological and cultural experiences. This leads to an oversimplified view that our mental lives can be completely explained by brain structure and chemistry.

“It seems as if the further neurology advances, the further the psyche recedes; psychopathology (…) explores the psyche to the limits of consciousness but finds at these limits no somatic process directly associated with such phenomena as delusional ideas, spontaneous affects and hallucinations” (Jaspers, 1997, p. 4).

In terms of historical significance, the clinical psychologist has been seen as having less status within the mental health context compared to the psychiatrist representing the medical doctor. Compared to the psychologist only being able to conduct psychotherapy, the psychiatrist has been able to do both psychotherapy and to prescribe medication. Lately, however, the continuous efforts of recovery-oriented psychiatry, social psychiatry, and community psychiatry have been able to revert the public eye toward the importance of interpersonal relationship and sociality regarding psychiatric issues and problems (Englander, 2018; Ferrarello, 2021; Brencio, 2024).

For this Research Topic in *Frontiers of Psychology*, we have addressed the historical and philosophical problems related to the subdiscipline called, *Theoretical and Philosophical Psychology*. As editors of this Research Topic, we have encouraged contributors to return to a phenomenological approach. The Research Topic, *Phenomenological psychopathology: who, what and how? An analysis of key figures, advancements and challenges* includes contributions that have addressed core philosophical issues relevant for the world of psychiatry and psychology.

Messas et al. give a detailed framework about the dialogue between phenomenology and psychopathology in the article entitled *Phenomenology yesterday, today, and tomorrow: a proposed phenomenological response to the double challenges of contemporary recovery-oriented person-centered mental health care*, where they argue that a dialectical synthesis of phenomenology's traditional twin roles in psychiatry (one science-centered, the other individual-centered) is needed to support the recovery-oriented practice that is at the heart of contemporary person-centered mental health care. The paper is divided into two main sections. Section I illustrates the different ways in which phenomenology's two roles have played out over three significant periods of the history of phenomenology in twentieth century psychiatry: with the introduction of phenomenology in Karl Jaspers' General Psychopathology in 1913; with the development a few years later of structural phenomenological psychopathology; and in the period of post-War humanism. Section II is concerned with the role of phenomenology in contemporary mental health. There has been a turn to phenomenology in the current period, in response to what amounts to an uncoupling of academic psychiatry from front-line clinical care. Corresponding with the two roles of phenomenology, this uncoupling has both scientific aspects and clinical aspects.

In recent explorations of phenomenological psychopathology, Rasmussen and Parnas (2024) propose that imagination anomalies in schizophrenic spectrum disorders (SSDs) stem from a fundamental self- or ipseity disorder. Their argument rests on a phenomenological model of consciousness, where each experience is articulated through ipseity based on its modality. They describe imagination as the representation of an absent object imbued with a sense of unreality, positing that imagination disturbances in SSDs signify a breakdown in this consciousness structure. This article extends their framework by integrating Marc Richir's work in Phantasia, Imagination et Affectivité (Richir, 2004), which presents an alternative consciousness model. Richir's approach: (a) differentiates phantasia from imagination, (b) incorporates affectivity into these processes, (c) situates imagination pathologies in failed empathic engagements, and (d) aligns these pathologies with psychoanalytic ideas of phantasm fixation.

Guardascione's article, *Selfhood and alterity: schizophrenic experience between Blankenburg and Tatossian*, offers a comparative analysis of two phenomenological views on schizophrenia. Blankenburg's concept of basal disturbance underscores the loss of natural self-evidence, emphasizing intersubjective disconnection as schizophrenia erodes rootedness in the social world, leading to derealization and depersonalization. Tatossian, by contrast, interprets schizophrenia as an intrasubjective disorder, disrupting the dual nature of transcendental subjectivity as both constitutive consciousness and reflective onlooker. Guardascione connects these interpretations to the ipseity-disturbance model, exploring their use of Husserlian phenomenology and deepening the understanding of schizophrenia through transcendental subjectivity and intersubjectivity.

Schizophrenia is also the focus of Martin's et al. work entitled *Relating movement markers of schizophrenia to self-experience—a mixed-methods study*. As many authors have emphasized (see for instance, Sheets-Johnstone, 1999) movement and our kinaesthetic abilities are fundamental in the process of self-development and in the knowledge of the environment. Thanks to the combination of qualitative and quantitative data, the authors suggest an association between movement markers and basic self-disorders, specifically in the domain of cognition, self-experience and bodily experiences. While movement marker manifestation is not precisely reflected in the individuals' descriptions of anomalous self- and body experience, there are more and more intense descriptions with increasing movement marker scores, when looking at specific experiences, such as hyper reflexivity.

Another disorder which has been at the center of many phenomenological reflections is depression: Frohn and Moltke Martiny, in the paper *The phenomenological model of depression: from methodological challenges to clinical advancements*, introduce the results of a phenomenological interview applied to 12 participants. They show how phenomenological interviews deal with the challenge of patho-description and how patho-description in depression conceals experiential nuances. Accordingly, they describe how people with depression pre-reflectively experience a variety of feelings, a type of agency, overly positive self-image, and relations in a hyper-social way. These descriptive nuances not only strengthen the phenomenological model of depression, but they also help advance the clinical work on depression.

A phenomenological approach appears to be useful also in distinguishing between different forms of depression. It is what is done in the article “*I was very sad, but not depressed”: phenomenological differences between adjustment disorder and a major depressive episode* by Zapata-Ospina et al. A descriptive phenomenological approach is used with in-depth interviews to four patients and the method proposed by Colaizzi to understand the experiences and reach the description of both disorders. While the MDE is described as an intense state of generalized shutdown of the subject's own life, with little response to events, the AD seems to be a dynamic reaction attributed to a stressful event, with high variability in the course of symptoms due to the dependence on such event, with the preserved hhat it will end.

Sánchez Guerrero and Wessing focus on the rate of depression in adolescence, and in the article *A phenomenologically grounded specification of varieties of adolescent depression* they show how phenomenology can be helpful to elaborate a fine-grained description of this clinical and experiential condition in that peculiar and difficult phase of life. Their study investigates the association between these types of depression typical of adolescence and the vicissitudes of personality development. In accounts given by youths diagnosed with depression during semi-structured interviews, they identify themes and examine their phenomenological centrality. Specifically, thanks to a qualitative analysis they differentiate three specifiers of adolescent depression and suggest an association between types of experiences and the trajectory of affected adolescents' personality development.

In examining the self-boundary in psychotic experiences within sociocentric cultures, Alphonsus et al. provide insights into how collective identities shape self-experience and boundary perception in schizophrenia. Moving beyond Western individualistic frameworks, this study reveals how psychotic experiences in collectivist cultures like Jaffna, Sri Lanka, are closely tied to social interactions. This work challenges the standard phenomenological psychiatric view, adding a critical cultural dimension to the intersubjective dynamics of psychosis and recovery.

Green and Shaughnessy make the point on the phenomenological debate about autism. *In Autistic phenomenology: past, present, and future* they review the emerging phenomenological work on autism (such as alongside a contemporaneous clinical phenomenology perspective) and representations of autistic experience from within the extensive literature (including life writing) from autistic people themselves. The result is an empirical autistic phenomenology, able to account both for the first-person experiences of autistic people and to a shared understanding.

Pantazakos and Vanaken's work, *Addressing the autism mental health crisis: the potential of phenomenology in neurodiversity-affirming clinical practices*, underscores the need for autism acceptance in therapeutic approaches, critiquing traditional methods that prioritize symptom reduction. They propose phenomenological psychology as a means to craft neurodiversity-affirming therapies, suggesting that these approaches could significantly improve mental health outcomes for autistic individuals by balancing acceptance of the “autistic self” with therapeutic goals.

Taipale's article, *Caught on the surface: tustin on autistic experience*, delves into Frances Tustin's (1981) theory, suggesting that sensory alterations, especially tactile, lie at the heart of autism. Tustin's work suggests that autistic individuals experience a collapse in experiential depth, which complicates interactions with both the world and others, impacting not only spatial perception but also symbolic and intersubjective connection.

In the paper *Incels, autism, and hopelessness: affective incorporation of online interaction as a challenge for phenomenological psychopathology*, Tirkkonen and Vespermann analyze the experiential connection between inceldom, self-reported autism, and hopelessness. By combining empirical studies on online incel communities with phenomenological and embodiment approaches on autism, hopelessness, and online affectivity, they analyze three interrelated aspects of online interactions in incel communities—worldview, bodily self-relation, and mutual dismissals—and examine how these elements contribute to the consolidation of the loss of significant life possibilities. This can shed light on the negative effects of specific online environments on autistic subjects.

Fukuda et al.'s *Obsessive–compulsive existential type: a dialectical-phenomenological approach* examines a condition which is usually overlooked in phenomenological psychopathology: the “obsessive–compulsive disorder.” By combining phenomenology and anthropology, they offer a description of the obsessive-compulsive existential type. They analyze this obsessive-compulsive lifeworld starting from the notion of anti-eidos as a diluting existential force, and they propose to enlarge this notion in its dialectical correlation with eidos (unifying existential force), representing the existential dialectic between transformation and permanence. The use of clinical cases and the theoretical regard lead to a detailed analysis of the different structures of subjectivity (such as temporality, embodiment etc.) in this peculiar condition.

Phenomenology is also used in the context of clinical emergency: in their paper entitled *Phenomenology of psychiatric emergency*, Goretti et al. takes into account life-threatening conditions and they argue that, even if until now neglected by phenomenological psychopathology, the emergency issue faces a clinical management challenge in which the phenomenological method becomes fundamental. The purpose of this manuscript is then to explore the phenomenological perspective of psychiatric emergencies. The manuscript is organized into four sections: the first deals with the encounter in clinical phenomenology, the second with the life-word of the crisis, the third with the atmosphere of emergency; finally, a final section on the importance of the phenomenological method for the clinician.

Wantoch's paper explores the phenomenon of anomalous experience, a term that encompasses what is often labeled as hallucination or, more broadly, the experiential dimensions of psychosis. She critically examines the prevailing clinical view, which frames anomalous experience as a “pathology of the mind,” and interrogates how this framing is intersubjectively felt and enacted. Drawing on Ratcliffe's (2017) account of intersubjectivity in relation to anomalous experience, Wantoch argues that psychiatric conceptualizations may inadvertently disrupt intersubjective processes for individuals undergoing such experiences. The pathologizing framework, she suggests, can shape relational dynamics in affectively charged ways that exclude individuals from shared meaning-making and social reality. Paradoxically, the very psychiatric framework intended to categorize and treat these experiences might also contribute to their emergence and intensification.

Wantoch challenges phenomenological psychopathology to reconsider its foundational assumptions, particularly its tendency to adopt psychiatric categories uncritically. She contends that the field must reflect on its own participation in shaping anomalous experience through conceptual and affective pathways. As an alternative, she advocates for beginning with the direct, lived experience of anomalous phenomena rather than with their presumed pathological status. In doing so, she points toward the development of a critical phenomenological psychopathology—one that acknowledges the co-constitutive role of clinical discourse, institutional practice, and interpersonal affect in shaping what counts as mental illness.

Lastly, Uljée's *The Unconscious and the Transcendental: Husserlian Phenomenology in Intersubjective Systems Theory* proposes that Husserl's phenomenology can ground intersubjective systems theory, a pivotal development in relational psychoanalysis. Uljée argues that phenomenology can clarify the intersubjective constitution of meaning while establishing a dynamic relationship between consciousness and the unconscious, thus bolstering the theoretical foundation of intersubjective systems theory.

Together, these works illustrate a broadened understanding of phenomenological approaches to psychopathology, highlighting the role of intersubjective, cultural, and developmental factors across various psychological conditions. They underscore the potential of phenomenology to deepen insights into complex mental health conditions and improve therapeutic practices. When diagnosing and treating mental health conditions, it's important to acknowledge how deeply a person's experiences, relationships, and social interactions shape their psychological development. Understanding mental health requires recognizing the continuous back-and-forth relationship between the brain and a person's environment (Fuchs, 2018). There is a crucial difference between describing someone's personal experience and diagnosing a mental disorder. Personal experiences are inherently subjective, while diagnoses require generalized criteria that can reliably predict outcomes. This led to a major shift in the 1980s, when the DSM-III introduced operational definitions. This approach was adopted to help clinicians reach more consistent diagnoses with less room for interpretation. However, while having standardized categories can be useful for grouping similar conditions, the oversimplified operational approach don't capture the full complexity of the experiences that people have in dealing with a psychological condition (Kendler and Parnas, 2012).

In editing this Research Topic our aim is to offer the readers a diverse Research Topic of contributions which crosscuts psychiatry, psychology and philosophy, and that can contribute to nourish a view on human suffering and behaving which cannot be inscribed only and mainly on a biomedical model. If *treating and curing* remain the priorities of every medical endeavor, *understanding* is the preliminary step toward these purposes and, as such, it needs to be grounded on a richer and diversified paradigm. In this, Jaspers' words can serve as a compass:

“In our view psychic life is an infinite whole, a totality that resists any consistent attempt to systematize it; much like the sea, we may coast along the shore, go far out into the deeps but still only traverse the surface waters. If we try to reduce psychic life to a few universal principles and seek comprehensive laws, we beg a question that cannot be answered. Where our theories may seem to have some kinship with the natural sciences, it is in the forming of tentative hypotheses, which we make for limited research ends only and which have no application to the psyche as a whole” (Jaspers, 1997, p. 17).

## References

[B1] Brencio F. (2024). Phenomenology, Neuroscience and Clinical Practice. Transdisciplinary Experiences. Cham: Springer Nature.

[B2] Englander M. (2018). Phenomenology and the Social Context of Psychiatry: Social Relations, Psychopathology, and Husserl's Philosophy. London: Bloomsbury.

[B3] Ferrarello S. (2021). The Role of Bioethics in Emotional Problems: A Phenomenological Analysis of Intentions. London: Routledge.

[B4] Fuchs T. (2018). Ecology of the Brain. Oxford: Oxford University Press.

[B5] Jaspers K. (1997). General Psychopathology (J. Hoenig and M.W. Hamilton, Trans.). London: Johns Hopkins University Press.

[B6] Kendler K. Parnas J. (2012). Philosophical Issues in Psychiatry II: Nosology. Oxford: Oxford University Press.

[B7] Rasmussen A. Parnas J. (2024). Imagination anomalies in schizophrenic spectrum disorders. Analysis based on a phenomenological modelof consciousness and ipseity. Front. Psychol. 15:1220099. 10.3389/fpsyg.2024.1220099

[B8] Ratcliffe M. (2017). Experiences of Depression: A Study in Phenomenology. Oxford University Press.

[B9] Richir M. (2004). Phénoménologie en esquisses. Paris: Éditions De Boeck Université.

[B10] Sheets-Johnstone M. (1999). The Primacy of Movement. Amsterdam: John Benjamins Publishing.

[B11] Tustin F. (1981). Autistic States in Children. London: Routledge.

